# Platelet and Plasma Phosphatidylcholines as Biomarkers to Diagnose Cerebral Amyloid Angiopathy

**DOI:** 10.3389/fneur.2020.00359

**Published:** 2020-06-12

**Authors:** Bettina M. Foidl, Herbert Oberacher, Josef Marksteiner, Christian Humpel

**Affiliations:** ^1^Laboratory of Psychiatry and Experimental Alzheimer's Research, Medical University of Innsbruck, Innsbruck, Austria; ^2^Institute of Legal Medicine and Core Facility Metabolomics, Medical University of Innsbruck, Innsbruck, Austria; ^3^Department of Psychiatry and Psychotherapy A, Hall State Hospital, Hall in Tirol, Austria

**Keywords:** Alzheimer's disease, cerebral amyloid angiopathy, platelets, biomarker, metabolomics, lipids, mouse model

## Abstract

Alzheimer's disease is a severe neurodegenerative brain disorder and characterized by deposition of extracellular toxic β-amyloid (42) plaques and the formation of intracellular tau neurofibrillary tangles. In addition, β-amyloid peptide deposits are found in the walls of small to medium blood vessels termed cerebral amyloid angiopathy (CAA). However, the pathogenesis of CAA appears to differ from that of senile plaques in several aspects. The aim of the present study was to analyze different lipids [phosphatidylcholines (PCs) and lysoPCs] in platelets and plasma of a novel mouse model of sporadic CAA (1). Our data show that lipids are significantly altered in plasma of the CAA mice. Levels of eight diacyl PCs, two acyl-alkyl PCs, and five lysoPCs were significantly increased. In extracts of mouse blood platelets, four diacyl and two acyl-alkyl PCs (but not lysoPCs) were significantly altered. Our data show that lipids are changed in CAA with a specific pattern, and we provide for the first time evidence that selected platelet and plasma PCs may help to characterize CAA.

## Introduction

Alzheimer's disease (AD) is a progressive neurodegenerative disorder in the brain. The major hallmarks are extracellular β-amyloid (Aβ) plaques, intracellular tau neurofibrillary tangles, cholinergic neurodegeneration, and cerebrovascular damage (2). In many cases, a comorbid cerebral amyloid angiopathy (CAA) is found, which is characterized by the accumulation of Aβ_40_ in the vessels. However, CAA is also present as an independent pathology without AD and can be characterized as a subform of vascular dementia (vaD) (3). The origin of AD, vaD, and CAA is still not fully understood. Approximately 95% of all CAA cases are sporadic, and only 5% are explained by a genetic background (2). More and more research suggests that vascular risk factors may play a role in the development of both sporadic CAA and AD (3–6).

At present, the diagnosis of AD and/or CAA combines psychological tests, brain imaging, and the analysis of four biomarkers (Aβ_40_, Aβ_42_, total tau, and phospho-tau-181) in the cerebrospinal fluid (CSF). For CAA especially, Aβ_40_ levels in CSF are investigated, as the values are lower in CAA compared to controls and AD patients (7). Furthermore, for CAA, the Boston criteria are used, which classify “definite CAA” in autopsy postmortem, “possible CAA” with brain imaging combined with clinical exclusions, and “probable and possible CAA” by tissue biopsy or the detection of multiple intracerebral hemorrhages (8, 9). However, the problem arises that the complete procedure is time consuming, and the collection of CSF is an invasive procedure. Moreover, in CSF samples, it is difficult to distinguish between AD and CAA or mixed forms of dementia (10, 11). Thus, the search for blood/plasma biomarkers is of great interest, as those biomarkers may offer a fast and non-invasive diagnostic method. In addition, platelets are of special interest, as they contain a high amount of the amyloid precursor protein (APP) and release approximately 90% of peripheral Aβ (mainly Aβ_40_) (12, 13). Recently, we and others have shown that lipidomics may offer a promising tool to diagnose AD not only in CSF (14), in plasma (15, 16), or in platelets (17) but also in saliva (18).

**Figure 1:**
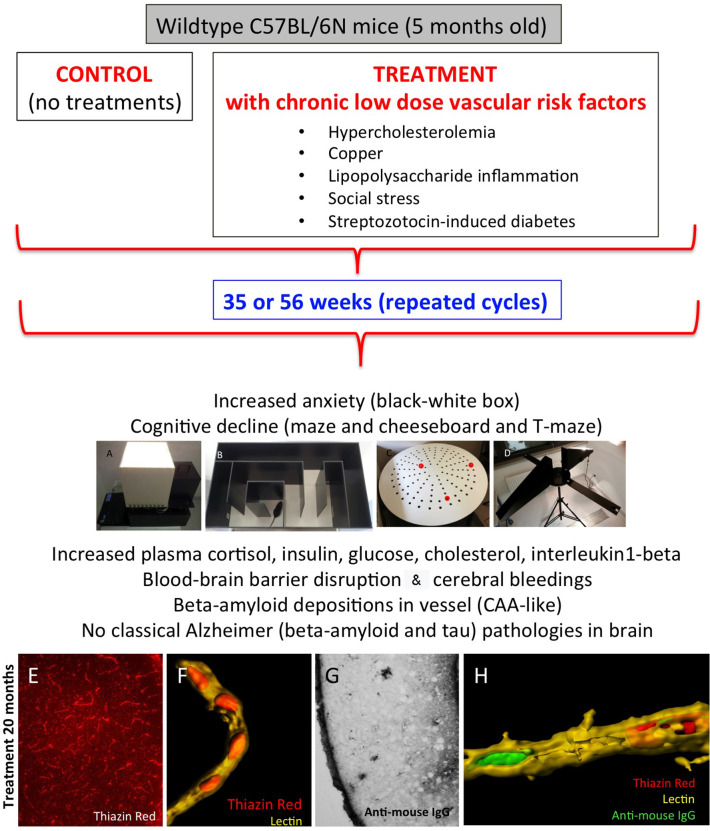
Characterization of the novel sporadic mouse model of cerebral amyloid angiopathy (CAA). Healthy 5-month-old C57BL/6N mice were treated for 35 or 56 weeks with different low-dose vascular risk factors (treatments) or without (controls). The behavior was tested all 6 weeks using a black–white box **(A)**, labyrinth maze **(B)**, cheeseboard maze **(C)**, and at the end of the experiment with a T-maze **(D)**. Mice were analyzed for plasma levels and brain vessels and AD-like pathologies. Note that these mice exhibit a severe pathology for CAA, as seen by thiazine red staining **(E)** in lectin + vessels **(F)** and blood–brain barrier disruptions as seen by anti–mouse IgG staining **(G)**. **(H)** a representative confocal microscopy staining of a lectin + vessel (yellow) with thiazine red inclusions (red) and anti–mouse IgG (green). For methodological details and details on the model, refer to our original publication (1).

Animal models are of great interest in studying mechanisms and potential treatments for CAA. In the last years, transgenic mouse models expressing the human APP have been developed. Many of these mouse models develop CAA in addition to senile plaques (19). Recently, we have generated a mouse model to study specifically CAA pathogenesis (1). Mice exposed to five vascular risk factors (hypercholesterolemia, copper in the drinking water, diabetes, inflammation, and social stress) showed drastic vessel pathology, cognitive decline, vascular bleedings, and the deposition of Aβ in the vessels (Figure 1) (1). A comparison in the expression pattern of lipids between the mouse model and AD patients can provide important information about the pathogenesis of CAA. We will use a well-established metabolomic lipid platform to identify changes in phosphatidylcholines (PCs), lysoPCs and sphingomyelins in plasma and platelet extracts. Specific changes in the lipid expression pattern can be helpful for diagnosis, course of disease, and treatment. Thus, in the present study, we aimed to measure plasma and platelet lipids in this CAA mouse model to differentiate and identify CAA-specific biomarkers.

## Methods

### Collection of Plasma and Platelets From Sporadic CAA Mouse Model

Sporadic CAA mice (*n* = 8) were generated as described in detail by us (1). All animal experiments were approved by the Austrian Ministry of Science and Research (BMWF-66.011/011_WF/V/3b/2015) and conformed to the Austrian guidelines on animal welfare and experimentation. Briefly, 5-month-old wild-type mice (C57BL/6N) were either not treated (controls) or treated with vascular risk factors for 35 or 56 weeks (Figure 1). Vascular risk factors contained 2% cholesterol food, copper in the drinking water (1 mg/L), streptozotocin (to induce diabetes; maximum dose 50 mg/kg), lipopolysaccharides (to induce inflammation; 1.25 mg/kg), and social stress (induced by changing the cage partners). Mice were anesthetized with 100 mg/kg ketamine and 10 mg/kg xylazine. Blood was taken directly from the heart and collected in EDTA tubes. Subsequently, the blood was centrifuged (10 min, 100 × *g*) at room temperature (RT) to obtain platelet-rich plasma (PRP). The supernatant was taken, and 500 nM prostaglandin (PGI2; Sigma, Vienna, Austria) was added. Plasma was centrifuged again (10 min, 400 × *g*, RT) to isolate platelets from PRP, and then the plasma supernatant and platelet pellets were frozen at −80°C until use.

### FACS Analysis

Fluorescence Activated Cell Sorting (FACS) analysis was performed as reported by us previously in detail (20). Immediately after isolation, two microliter mouse platelets were incubated with antibodies against immunoglobulin G1 (IgG1)–fluorescein isothiocyanate (FITC) (BD 555748) or CD41/CD61-FITC (LeoF2, Emfret M025-1) or CD42a-FITC (XiaB4, Emfret M051-1) or CD42b-FITC (XiaB2, Emfret M043-1) in 50 μL FACS buffer [2 mM EDTA, 0.5% FCS ad 100 mL phosphate-buffered saline (PBS), pH 7.1] for 30 min at 4°C in dark. All samples were centrifuged at 300 × *g* for 10 min, and the pellets were resuspended in 100 μL of FACSFlow (BD FACSFlow, Erembodegem, Aalst, Belgium). FACS analysis was instantly performed with a BD FACScan.

### Targeted Metabolomic Analysis of Plasma and Platelets

The endogenous metabolites were analyzed with a targeted quantitatively and qualitatively controlled metabolomics assay by using the AbsoluteIDQ p150 Kit (Biocrates Life Science AG, Innsbruck, Austria). This validated assay allows the quantification and comprehensive identification of 163 endogenous metabolites including among others 77 PCs (PC aa = diacyl x:y; PC ae = acylalkyl x:y) and 40 acylcarnitines (Cx:y). The AbsoluteIDQ p150 Kit was performed according to the manufacturer's instructions as reported by us (16, 17). In short, 10 μL of sample mixture was pipetted onto filter spots suspended in the wells of a 96-well filter plate. The filter plate was fixed on top of a deep-well plate serving as a receiving plate for the extract later on, that is, a combi-plate structure. After drying under a nitrogen stream for 30 min, 50 μL of a 5% phenylisothiocyanate solution was added to enable derivatization of amino acids. After 20 min of shaking and nitrogen drying, 300 μL of 5 mM ammonium acetate in methanol was added to the wells. After 30 min of incubation, the combi-plate was centrifuged to move the extracts into the lower receiving deep-well plate, which was then detached from the upper filter plate. After adding another 300 μL of 5 mM ammonium acetate in methanol to the extracts and briefly shaking, the plate was placed in the autosampler of the flow injection analysis (FIA)–tandem mass spectrometry (MS/MS) system for analysis. The FIA-MS/MS system consisted of a Knauer K-1001 LC pump (Knauer, Berlin, Germany), a CTC-PAL HTS9 autosampler (CTC Analytics AG, Zwingen, Switzerland), and a QTrap 3200 mass spectrometer (Sciex, Toronto, Ontario, Canada). The injection volume was 30 μL. The flow rate was set to 30 μL/min. Metabolite concentrations (μM) were automatically calculated by the MetIDQ software package part of the AbsoluteIDQ p150Kit.

### Western Blot Analysis

Western blot analysis was performed as previously described by us (21). Platelet samples (−80°C) were thawed and tubes dissolved in 100 μL ice-cold PBS containing a protease inhibitor cocktail (P-8340; Sigma). Samples were then sonicated using an ultrasonic device, centrifuged at 14,000 × *g* for 10 min at 4°C; the extracts were denatured (10 min, 70°C), and 18 μg was loaded onto 10% *bis*–*tris* SDS-polyacrylamide gels (Thermo Fisher Scientific, Vienna, Austria), separated for 35 min at 200 V and finally electrotransferred to nylon-PVDF Immobilon-PSQ membranes for 20 min at 30 V in 20% methanol blotting buffer. Next, blots were blocked for 30 min in blocking buffer; incubated with primary antibody against APP (Abcam ab32136, 1:2,000, Cambridge, UK), or CD41 (Abcam ab63323, 1:2,000), or actin (1:1,000, A2066; Sigma, Vienna, Austria) at 4°C overnight; washed; and then incubated in alkaline phosphatase–conjugated anti–rabbit IgG for 30 min. After washing, bound antibodies were detected using an enhanced chemiluminescence system and visualized by using a cooled CCD camera (SearchLight; Thermo Fisher Scientific).

### Statistical Analysis

Statistical analysis was performed with analysis of variance (ANOVA) and a subsequent Fisher least significant difference (LSD) *post-hoc* test and comparing controls vs. treatments. Statistical results were considered significant at *p* < 0.05.

## Results

### Lipids in Plasma of CAA Mice

Approximately 100 lipids were determined in the plasma of well-characterized CAA mice and compared to control mice (Table 1). Levels of eight aaPCs (PCaaC30:2, PCaaC34:1, PCaaC36:1, PCaaC38:4, PCaaC38:5, PCaaC38:6, PCaaC40:4, PCaaC40:6) and two aePCs (PCaeC38:0, PCaeC40:4) were significantly elevated. Five lysoPCs were significantly enhanced compared to the controls (lysoPC C16:0, lysoPC C16:1, lysoPC C18:0, lysoPC C18:1, lysoPC C20:4).

**Table 1:** Plasma levels of selected lipids altered in CAA mice.

		**Controls (56 weeks)**	**Sporadic CAA (56 weeks)**	***p*-value**
*n*		8	8	
lysoPC	C16:0	138 ± 5	193 ± 11	0.0005***
	C16:1	4 ± 0	6 ± 0	0.001***
	C18:0	67 ± 3	120 ± 13	0.001***
	C18:1	34 ± 2	58 ± 5	0.0004***
	C20:4	40 ± 3	69 ± 8	0.006**
PCaa	C30:2	2 ± 0	4 ± 0	0.002**
	C34:1	54 ± 5	74 ± 4	0.01**
	C36:1	11 ± 1	19 ± 1	0.0003***
	C38:4	67 ± 4	118 ± 13	0.003**
	C38:5	21 ± 2	31 ± 2	0.002**
	C38:6	58 ± 6	86 ± 4	0.002**
	C40:4	0.9 ± 0.08	1.4 ± 0.1	0.002**
	C40:6	20 ± 1	38 ± 5	0.002**
PCae	C38:0	1 ± 0	2 ± 0	0.01**
	C40:4	1.03 ± 0.08	1.5 ± 0.1	0.005**

Significantly altered lipids [phosphatidylcholines (PCs)] in plasma of mice with sporadic CAA (treated for 56 weeks) compared to same age-matched controls. Values are given as mean ± SEM (in μM); n represents the number of animals or patients per group. Statistical analysis was performed with ANOVA with a Fisher LSD post-hoc test (**p < 0.01; ***p < 0.001). PCaa, diacyl x:y; PCae, acylalkyl x:y.

### Lipids in Platelets of CAA Mice

**Figure 2:**
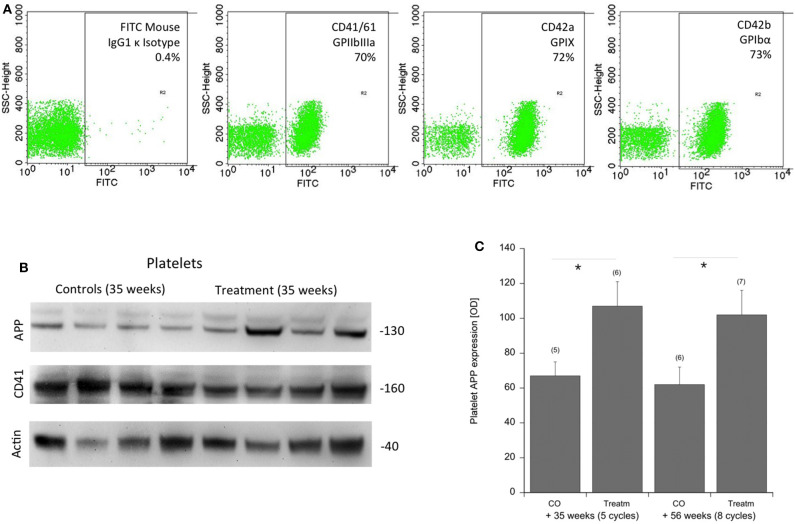
Characterization of platelets by FACS and APP expression in the mouse CAA model. **(A)** Platelets were characterized by FACS analysis for CD41/61 GPIIbIIIa, CD42a GPIX, CD42b GPIbα compared to an IgG control. **(B)** Amyloid precursor protein (APP) was measured by Western blots and showed a 130-kDa protein. Actin and CD41 served as controls. Size markers are given as kDa. **(C)** Statistical analysis was performed by ANOVA with a subsequent Fisher LSD *post-hoc* test (*p < 0.05). Note an increased expression of APP in platelets of sporadic CAA mice in both treatment groups at 35 and 56 weeks of age (Treatm) compared to age-matched controls (CO). Values are given as mean ± SEM optical density. Numbers in parenthesis show numbers of animals.

Approximately 100 lipids were determined in the platelets of well-characterized CAA mice and compared to control mice (Table 2). Platelets were evaluated by FACS analysis for CD41/61, CD42a, and CD42b (Figure 2A). Levels of six PCs (PCaaC36:5, PCaaC38:0, PCaaC38:5, PCaaC38:6, PCaeC36:1, PCaeC40:2) were significantly changed in platelet extracts taken from 56 week-old sporadic CAA mice, compared to age-matched controls (Table 2). As a positive control, APP was analyzed in isolated platelets and compared to CD41 and actin (Figure 2B). Western blot analysis showed a 130-kDa APP protein, and its expression was significantly higher in the CAA mice compared to the controls (Figure 2C), both in the 35 and 56 week treatment groups.

**Table 2:** Platelet levels of selected lipids altered in CAA mice.

		**Controls (56 weeks)**	**Sporadic CAA (56 weeks)**	***p*-value**
*n*		8	8	
PCaa	C36:5	0.02 ± 0.005	0.06 ± 0.01	0.01**
	C38:0	0.17 ± 0.01	0.08 ± 0.009	0.0002***
	C38:5	0.09 ± 0.03	0.3 ± 0.06	0.003**
	C38:6	0.14 ± 0.04	0.48 ± 0.11	0.01**
PCae	C36:1	0.14 ± 0.008	0.07 ± 0.01	0.0002***
	C40:2	0.05 ± 0.004	0.02 ± 0.0003	0.0001***

Significantly altered lipids [phosphatidylcholines (PCs)] in isolated platelets of mice with sporadic CAA (treated for 56 weeks) compared to the same age-matched controls. Values are given as mean ± SEM (in μM per 1 mg platelets); the n represents the number of animals or patients per group. Statistical analysis was performed with ANOVA with a Fisher LSD post-hoc test (**p < 0.01; ***p < 0.001). PCaa, diacyl x:y; PCae, acylalkyl x:y.

## Discussion

In the present study, we examined lipid levels in the plasma and platelets taken from a well-characterized novel mouse model of sporadic CAA. Our data show that 15 plasma lipids and 6 platelet lipids may help to characterize CAA.

### Pathology of Sporadic CAA

We recently characterized a novel mouse model of sporadic pure CAA pathology without AD pathology (Figure 1) (1). In the human form, CAA mostly comes along with a concurrent AD pathology, and the general problem arises that the two pathologies cannot be discriminated easily. Because CAA is a disease of the elderly, it overlaps with a variety of cardiovascular risk factors (e.g., hypertension, diabetes, hypercholesterolemia) that could contribute to CAA pathology. However, there is a strong suggestion that platelets contribute to the onset of CAA and AD (22). Specifically, in CAA dysfunctional platelets may play a crucial role. We hypothesize that due to lesions in brain vessels occurring over years, it also comes to a blood–brain barrier disruption and furthermore to vascular bleedings. These bleedings can cause dysfunction and overactivation of platelets. Subsequently, a production and release of Aβ and deposition of Aβ_40_ and possibly Aβ_42_ may cause CAA (22). Therefore, it is supposed that platelets and a preceding CAA pathology may be the first stage before progression to the AD pathology, and platelets may serve as putative biomarkers.

### Plasma Lipids

Recent data suggest lipid measures in plasma may produce detectable signatures in AD patients. A groundbreaking work has been published some years ago, where it has been postulated that a set of 10 lipids (C3, lysoPCaC18:2, PCaaC36:6, C16:1-OH, PCaaC38:0, PCaaC38:6, PCaaC40:1, PCaaC40:2, PCaaC40:6, and PCaeC40:6) from peripheral blood predicted the conversion to mild cognitive impairment (MCI) or AD within a 2 to 3 year timeframe with >90% accuracy (15). However, this study was very enthusiastic, and so far, the pattern of these 10 lipids could not be reproduced from other laboratories, and also this set did not go into routine analysis. We ourselves could not find the same pattern of lipids but found that the ratio of PCs to lysoPCs (PCaaC34:4 and lysoPCaC18:2) in plasma differentiated healthy controls from patients with AD and MCI (16). Very recently, a study showed that three serum lipids [SM(OH)C24:1, SMC24:0 and PCaeC44:3] differentiated MCI and early-stage AD patients (23). So far, no data have been published for lipids in CAA. Our data show for the first time that in a novel mouse model of sporadic CAA 15 plasma lipid metabolites are altered, which may distinguish CAA from the human AD pathology.

### Platelet Lipids and APP

It has been well-established that platelets play a major role in the progression of AD and CAA (22). Indeed, we and others have shown that platelets from an AD mouse brain are able to damage healthy brain vessels (20). It is well-known that platelets release Aβ into the blood, where it may play a role in blood clotting (24). Interestingly, platelets contain a high amount of APP, and it has been shown that the APP expression is altered in AD patients (25, 26). However, so far, these findings have never entered routine analysis, as the APP expression and AD pathology are very heterogeneous with a high variance. In the present study, we confirm the expression of APP in platelets but show that the APP expression is also altered in a sporadic CAA mouse model. While this is an important control experiment to show the physiological role of platelets, it also shows for the first time that platelet APP is altered in CAA without any AD pathology. This also further strengthens the hypothesis that platelet pathology contributes to vessel damage and subsequent deposition of plaques and AD progression (22).

In a previous study, we have already demonstrated that the lipid metabolism is altered in platelets of MCI and AD patients (17). We showed that soluble platelet PCaeC40:4 can be used as a marker for AD in platelets (17). However, it was very difficult to diagnose pure forms of CAA in humans. Thus, by generation of this mouse model of pure CAA, we are in a position to characterize the pattern of the platelet lipidom and to directly point to putative biomarkers in human CAA. Our data suggest that the lipidomic examination of platelets may allow diagnosing early changes of CAA. As it is very difficult to diagnose pure CAA in humans, our data may provide a differential diagnostic pattern, which could be useful to differentiate CAA in humans. The pathophysiology of CAA in humans is a complex process that is triggered by various risk factors, including aging, hyperlipidemia, hypertension, and diabetes. Thus, our CAA mouse model mimics this aspect of AD pathophysiology. Nevertheless, mice also display major genetic and physiological differences compared to humans.

### Limitations of the Study

Definitely, this study had some limitations. (a) A limitation of our mouse model is that despite developing specific changes, they do not progress to advanced stages that are observed in humans. Consequently, no spontaneous plaque rupture is observed in our mouse model. Furthermore, one evident difference between mice and humans resides in the lipoprotein metabolism. Mice are considered as a high-density lipoprotein (HDL) model because most of the cholesterol is transported in HDL particles, and not in low-density lipoprotein (LDL) as in humans. However, a recent study showed that the mouse exhibits protein diversity across the LDL and HDL size ranges that are generally similar to those in humans (27). (b) The question arises how mouse models correlate with humans. In neurobiology research, we all rely on animal models to study human diseases. Transgenic mouse models are well-known AD models, but do not exhibit a sporadic origin (1). No mouse models for sporadic AD have yet been reported. We (1) recently developed a mouse model of sporadic CAA without any AD pathology, which mimics some aspects of CAA. (c) As mentioned, all these models only partly reflect a full pathology of a disease. In our model, we exposed wild-type mice chronically to five vascular risk factors, which caused a vessel pathology. But definitely, we do not know which risk factors cause CAA in humans, and again our model only partly reflects a human-related CAA.

## Conclusion

Taken together, our results show that 15 plasma lipids and 6 platelet lipids may help to characterize CAA. Our model provides the basis for further studies in humans, where the results can be compared and pathophysiological changes in the plasma and platelets could be a useful tool for the early diagnosis of CAA.

## Data Availability Statement

The datasets generated for this study are available on request to the corresponding author.

## Ethics Statement

The animal study was reviewed and approved by Austrian Ministry of Science.

## Author Contributions

BF developed the sporadic CAA mouse model, isolated and analyzed platelets, evaluated all data, and wrote the manuscript. HO performed the lipidomic analysis. JM designed the study and wrote the MS. CH financed the study, designed the project, and wrote the manuscript.

## Conflict of Interest

The authors declare that the research was conducted in the absence of any commercial or financial relationships that could be construed as a potential conflict of interest.
